# Transactions of the Pennsylvania Association of Surgeon Dentists

**Published:** 1846-03

**Authors:** 


### ARTICLE III.

*Transactions of "The Pennsylvania Association of Surgeon Dentists,"* held in the City of Philadelphia, December 15th, 1845.

IN accordance with a call signed by a number of practicing dentists, resident in Pennsylvania, a convention, composed of members of the profession, was held in Philadelphia, December 15th, 1845, for the purpose of taking the requisite steps for the formation of a State Dental Association.

On motion of Dr. J. D. White, Dr. Eli Parry, of Lancaster, was called to preside over the Convention, and J. M. Harris, of Philadelphia, was appointed Secretary.

After the Convention was called to order, the list of the names of those individuals, to whom the circulars, calling the Convention, had been addressed, was read by the Secretary, when the following gentlemen were found to be present:—Dr. Eli Parry, *Lancaster*, Dr. G. A. Planton, C. C. Williams, Dr. R. Arthur, J. M. Harris, Stephen T. Beale, S. Stockton White, Wm. D. White, F. Reinstein, J. D. Moore, Mr. Ingram, —— Foster, Jr., A. R. Johnson, Dr. J. D. White, Thomas Wardle, E. M. Neal, H. M. Porter, A. M. Asay, T. W. Evans, Sam'l L. Mentzer, James O'Neil, S. I. Dickey, T. L. Buckingham, W. J. Mullen. *By Proxy:* Dr. J. Locke, *Bellefonte*, S. Robert Dickson, *Schuylkillhaven*, Charles H. Bressler, James Parry, *Lancaster*.

Upon a call from the chair, written communications to the Convention from the following gentlemen were received and ordered to be filed: Dr. J. Locke, of *Bellefonte;* S. Robert Dickson, Schuylkillhaven; Charles H. Bressler, Bellefonte, James Parry, Lancaster.

The rules and order of business of the Lancaster City and County Medical Society were then adopted for the government of the Convention in its deliberations.

It was then, on motion of Dr. J. D. White,

*Resolved*, That, in the opinion of this Convention, the time has arrived, when it is expedient to organize an association of dental surgeons, in this State, for the purpose of cultivating the science of dental surgery, and of elevating and sustaining the character of the profession.

On motion of J. D. Moore, a committee of five was then appointed to draft a constitution, for the organization of the proposed society. The committee consisted of the following gentlemen :—Dr. J. D. White, Dr. G. A. Planton, W. J. Mullen, C. C. Williams and Stephen T. Beale.

After a short absence the committee returned and reported to the convention a preamble and constitution, which, after some trifling amendments, were adopted, as follows :

PREAMBLE.

Whereas, we, the undersigned dental surgeons of the state of Pennsylvania, have agreed to associate ourselves for the purpose of promoting the honor, character, and interests of the dental profession, so far as they are consistent with morality, and the general good of mankind ; and whereas, we are desirous of acquiring the rights and immunities of a body politic and corporate, for the furtherance of this honorable and legal purpose.

Now, therefore, we do hereby associate ourselves, under the following Constitution, for the purposes therein set forth :

### CONSTITUTION.

ARTICLE I.—*Of the Name.*

This Society shall be known by the name, style and title, of the Pennsylvania Association of Dental Surgeons.

ARTICLE II.—*Of the Objects.*

The objects of this society shall be : to cultivate the science of dentistry, and all its collateral branches, to elevate and sustain the professional character of dentists, and to promote amongst them mutual improvement, social intercourse and good feeling.

ARTICLE III —*Of the Officers.*

The officers of this society shall consist of a President, two Vice-presidents, a Recording and a Corresponding Secretary, an Examining Committee, a Treasurer, and such other officers as shall be designated by the by-laws, which officers shall be chosen annually from among the members of the society.

ALTICLE IV.—*Of its Powers.*

This society shall have power to make, have and use one common seal, with such device and inscription as it shall think proper: the same to break, alter and renew at its pleasure; and it shall have power and authority to make rules, by-laws and ordinances for its regulation, and to do every other act and thing needful for the government and support of the affairs of the said society, provided, always, that the said rules, by-laws and ordinances, or any of them, be not repugnant to this constitution; the constitution of the United States, or to the constitution and laws of this commonwealth.

ARTICLE V.—*Of Certificates.*

This society shall have power to grant to members and honorary members, in the manner and form prescribed by the by-laws, such certificate, under its common seal, as may authenticate and perpetuate the memory of such membership.

ARTICLE VI.

In case a dissolution of the society shall at any time be proposed, a meeting shall be called, specially, for that purpose, and the consent of three-fourths of the members present shall be required to effect it. Should the society be thus dissolved by its own act, the property belonging to it shall be sold by order of the President, or any three members, who shall first obtain authority from a majority of the surviving members. The proceeds of such sale shall be equally divided among said survivors.

ARTICLE VII.

This constitution may be altered and amended with the consent of two-thirds of the members present; the proposition stating the amendment, in writing, to be submitted by five members at one stated meeting and lie over until another stated meeting.

ARTICLE VIII.

The time and manner of holding meetings shall be regulated by the by-laws.

ARTICLE IX.

No misnomer of the said corporation shall defeat or annul any gift, grant, devise, or bequest, to, or from, the said corporation; provided the interest of the parties shall sufficiently appear on the face of the gift, grant, or other writing, whereby any estate or other interest was intended to pass to or from the said corporation.

On motion of J. M. Harris, the convention went into election for President of the proposed association, which resulted in the choice, for one year, of Dr. G. A. Planton.

It was here suggested, that, as it was possible that some who desired to take part in the proceedings of the convention, might have been unavoidably detained from the morning session, it would, perhaps, be more seemly, and prevent cavil, to adjourn till three o'clock, before entering upon the election of the rest of the officers, so as to allow any so disposed, who were not then present, to come forward.

Dr. Planton here rose, and said that he would greatly prefer that his election, as President of the Association, should be reconsidered, as he saw the propriety of the present adjournment. The convention, however, would not agree to reconsider his election.

The convention then adjourned, to meet at three o'clock, P. M.

*December* 15*th,* 1845, 3 *o'clock,* P. M.

Convention called to order by the Chairman.

Minutes of the morning session read and adopted.

The election of officers was then resumed, and continued till it resulted in the choice of the following gentlemen, to act in their different capacities for one year:

DR. ELI PARRY, 1*st Vice-president.*
STEPHEN T. BEALE, 2*d Vice-president.*
C. C. WILLIAMS, *Recording Secretary.*
DR. R. ARTHUR, *Corresponding Secretary.*
F. REINSTEIN, *Treasurer.*

It was then suggested, that, as the Examining Committee was one of such great importance to the Association, it would be more advisable to defer balloting for the purpose of filling it, until the Association was fully formed, which was agreed to.

It was then, on motion of Dr. J. D. White,

*Resolved,* That the members of the society now forming, be elected from this convention, individually, by ballot; that the candidate be required to absent himself from the room whilst the election is in progress; and that two-thirds of the whole number of votes be necessary to an election.

Whereupon the balloting took place, and resulted in the election of the following gentlemen:

James M. Harris, Wm. R. White, J. S. White, J. D. Moore, A. R. Johnson, Dr. J. D. White, Thomas Wardle, E. M. Neal, H. S. Porter, A. M. Asay, T. W. Evans, Samuel L. Mintzer, T. L. Buckingham, W. I. Mullen, James O'Neal, S. J. Dickey, W. H. Clark, C. L. Munns, Charles Moore, James O. Ely, *Philadelphia;* Dr. James Locke, Charles H. Bressler, *Bellefonte;* James Parry, *Lancaster;* S. Roberts Dickson, *Schuylkillhaven.*

The duties of the convention having terminated, it then adjourned, *sine die.*

The President elect of the Association was then conducted to the chair, and the duties of Recording Secretary assumed by C. C. Williams, Secretary elect.

The Association was then called to order by the President, who made some remarks appropriate to the occasion, and was followed by several other gentlemen, upon topics of interest to the Association.

On motion of J. M. Harris, a committee was appointed, for the purpose of drafting a code of By-laws, for the government of the Association; it was composed of the following gentlemen: J. M. Harris, Dr. Eli Parry, and Dr. J. D. White.

The committee, after a short absence from the room, reported a code of By-laws, which were read; after which the Association adjourned till Tuesday, 16th Dec., 10 o'clock, A. M.

*Tuesday, Dec. 16th, 10 o'clock,* A. M.

Adjourned meeting of the Association. The President in the chair. The Association called to order.

Minutes of the afternoon session of the convention, and those of the first session of the Association were read and adopted.

On motion of Dr. Eli Parry, who vouched for his qualifications, moral standing, &c. James O. Ely was duly elected a member of the Association.

The by-laws were then re-read, each article considered separately, amended, and adopted as follows:

### BY-LAWS.

### ARTICLE I.—*Of the President.*

The President shall preside at all meetings, keep order, state and put questions, regulate debates, and sign all orders on the Treasurer, duly passed by the society. He shall have power to call meetings, at his own discretion; and it shall be his duty to call them when requested, in writing, by five members of the society. In the call for a special meeting, he shall state its objects; and at the meeting, he shall not allow any other business to be transacted. He shall not discuss any question, while in the chair, unless it be a question of order. He shall have no vote, except where his vote may be necessary to decide a question, upon a call for the yeas and nays, or upon a ballot.

### ARTICLE II.—*Of the Vice-president.*

One of the Vice-presidents, in the absence of the President, shall assume all the duties of that officer, and, in the absence of all these officers, a chairman pro tem, shall be appointed *viva voce.*

### ARTICLE III.—*Of the Recording Secretary.*

The Recording Secretary shall keep accurate minutes of the proceedings of the society, and, after their approval, record them in a book provided for that purpose. He shall call the roll, read documents and attest all bills signed by the President. He shall keep a list of the members and honorary members on which he shall note the date of the election, and place of residence of each, the time of death, resignation or loss of membership. He shall receive and preserve all books and papers belonging to the society, not appertaining to the duties of other officers, and provide certificates of membership. He shall cause all by-laws, actually in force, to be inserted in a book, provided for that purpose, which shall be accessible to members, at every meeting of the society, and he shall perform such other duties as usually appertain to his office. He shall give notice of meetings, as directed by the President, or the society, and furnish the chairman of committees with copies of the minutes of their appointment. When absent he shall send the minute book to the meeting, and a secretary, pro tem., shall be appointed *viva voce.*

He shall deliver up to his successor all books and papers, belonging to the society, which may be in his hands.

ARTICLE IV.—*Of the Corresponding Secretary.*

The Corresponding Secretary shall give notice to all officers and members of their election, and manage all matters of correspondence, unless otherwise ordered by the society. He shall copy, into a book, provided for that purpose, all letters which he may write, officially, and keep a regular and uniform file of all those which he receives, and report at each stated meeting. He shall deliver up to his successor the books and papers belonging to the society.

ARTICLE V.—*Of the Treasurer.*

The Treasurer shall collect and receive all the monies due the society, and pay all orders, properly signed and attested, and these orders shall be his vouchers for his expenditures. He shall present a balanced account annually, at the stated meeting in October, and whenever called upon by the society; and shall deliver up to his successor all the books, papers, and funds, in his hands, belonging to the society, and give satisfactory security for the faithful performance of said duties.

ARTICLE VI.—*Of Members.*

This society shall consist of active and honorary members.

ARTICLE VII.—*Of Membership and Examining Committee.*

Any person, applying for membership, shall be twenty-one years of age, of good moral character, and have received a liberal English education. Such candidate shall apply, in writing, to the chairman of the Examining Committee, who shall have power to call the committee together, at such time and place as may suit the convenience of the parties, when the candidate shall submit to an examination by said committee, on the following branches of science: viz. Surgical and Mechanical Dentistry, General Anatomy, and Special Dental Anatomy, Physiology and Dental Pathology, Dental Therapeutics and Materia Medica, Theoretical Chemistry and Dental Hygiene. And if his qualifications, upon such examination, be satisfactory to a majority of said committee, they shall propose him to the society as worthy of membership, which proposition shall lie over till the next stated meeting, at which he shall be balloted for; two-thirds of all the votes given shall be necessary for his election. Those who have received diplomas, or certificates, from a respectable dental college or association (recognized as such by this society,) may be admitted to membership without an examination. No person elected shall have membership until he shall have paid his initiation fee, and subscribed to the constitution, and if he should omit the payment and signing above mentioned, for one year, his election shall be void.

ARTICLE VIII.—*Of Dues.*

The initiation fee, for members, shall be *ten dollars*, payable at or before the signing of the constitution. The annual contribution shall be *two dollars*, payable at the annual stated meeting. Any member who shall neglect to pay his annual contribution, two successive years, shall forfeit his membership.

ARTICLE IX.—*Of Honorary Members.*

Any gentleman, of good moral character, who has distinguished himself by any useful dental publication, on important improvement or discovery in science, shall be considered eligible for honorary membership. Nominations for such membership may be made in writing, by any two members of the society, stating the claims of said nominee, to such distinction, after which he may be balloted for; two-thirds of all the votes given shall be necessary for election.

ARTICLE X.—*Privileges of Members.*

Each member shall be entitled to debate, and vote on all questions agitated in the society, and be eligible to any office in the gift of the society. Each honorary member shall be entitled to a seat in all meetings of the society, and have the privilege of debating all questions not involving pecuniary expenditure. Every member and honorary member, shall be entitled to receive a certificate, according to the annexed form, having the seal of the society affixed thereto, and signed by the officers of the Association.

*Form of Certificate.*

The Pennsylvania Association of Dental Surgeons, instituted for the improvement of dental science, the promotion of social intercourse among dental practitioners, for supporting the character of the dental profession, reposing confidence in the knowledge, skill and integrity of A—— B—— have associated him as —— member thereof.

In witness whereof, we have unto annexed the names of the proper officers, and the seal of the corporation, this —— day of

ARTICLE XI.

The above certificate shall be the property of the society, to be returned on the expulsion of a member, or honorary member, and in case of failure to return the certificate after due notice, he shall be subject to public exposure by the society.

ARTICLE XII.

The resignation of no member shall be accepted until he shall have discharged all his dues to the society.

ARTICLE XIII.—*Of Expulsions.*

Any member, or honorary member, may be impeached by three members, for contravening the laws of this society, for malpractice, or other gross misconduct. The member so impeached shall have transmitted to him a written copy of the impeachment, with notice of the time of hearing, before a committee, appointed for that purpose, which time shall not be less than one month after the member shall have received said notice; then, if the report of said committee sustain the impeachment, the society, at a stated meeting, may, by ballot, suspend, or expel, said member by a vote of two-thirds of all the votes given. After a member, or honorary member, has been expelled, professional intercourse, between him and the rest of the members, shall cease.

ARTICLE XIV.—*Of Meetings.*

*Sec.* 1. Five active members shall be necessary to constitute a quorum, for the transacting of any business at a meeting of the Society.

*Sec.* 2. This society shall hold four stated meetings, annually, at such place as the society may from time to time direct, and in the following order, viz. first stated annual meeting on the first Tuesday in October; second stated meeting on the second Tuesday in December; third stated meeting on the third Tuesday in February; and the fourth stated meeting on the third Tuesday in April.

ARTICLE XV.—*Of Meetings and Rules of Order.*

Sec. 1. At stated meetings, the following shall be the order of business; the roll shall be called, and dues collected.

2d. The minutes of the preceding meeting shall be read, for approval or correction.

3d. Officers and committees shall report.

4th. Stated business of the meeting.

5th. Unfinished and deferred business.

6th. Election of members.

7th. Election of officers.

8th. Nominations for membership.

9th. New business.

10th. Communications in writing from those having no membership.

11th. Oral communications from those having no membership.

12th. Observations thereon.

13th. Written communications from those having membership.

14th. Oral communications from those having membership.

15th. Observations thereon.

*Sec.* 2. No question shall be considered open for discussion, except when brought forward by motion, duly made and seconded, and then distinctly stated by the presiding officer. The names of the mover and seconder of each motion to be entered on the minutes.

*Sec.* 3. When an individual speaks, he shall stand up, address himself to the presiding officer, and confine himself strictly to the question under consideration.

*Sec.* 4. No person shall be interrupted, whilst speaking, except by a call to order.

*Sec.* 5. The presiding officer shall decide all calls to order unless an appeal be made to the society; the vote upon which shall be taken without debate.

*Sec.* 6. No person shall be allowed to speak more than twice upon the same question, without previously asking, and obtaining, leave from the members present.

*Sec.* 7. Whilst any question is under consideration, no motion shall be received, excepting to divide the question, to amend, to postpone, to refer to a committee, or to adjourn.

*Sec.* 8. Motions for a division of the question, for its postponement, or reference to a committee, and for adjournment, shall be always determined without debate.

*Sec.* 9. A motion to adjourn shall take precedence of all others; a motion for postponement shall preclude commitment; that for commitment shall preclude amendment, or a decision on the original question.

*Sec.* 10. The mover, with the consent of the seconder, may withdraw any motion, previously to its amendment, commitment, or to the question upon its final passage, being put by the presiding officer.

*Sec.* 11. No question shall be reconsidered, excepting on the motion of two persons, who voted in the majority, submitted at the meeting at which the same was discussed.

*Sec.* 12. A motion that has been negatived, cannot be again brought forward at the same meeting, excepting on a motion to reconsider.

*Sec.* 13. When a blank is to be filled, the question shall be first taken on the largest sum, greatest number and remotest period.

*Sec.* 14. Two members may demand the yeas and nays on any question which is not required to be decided by ballot, and have them entered upon the minutes. The presiding officer, in such cases, shall always vote last.

*Sec.* 15. The students of members, and associates, shall be admitted to the sittings of the society, on producing a certificate from their preceptors.

*Sec.* 16. A stranger may be introduced by any member to the sittings of the society, by the consent of the President, and join in discussions by a vote of the majority.

ARTICLE XVI.—*Of Committees.*

Special committees shall be appointed in the following manner, excepting when otherwise ordered by a special vote of the society. The presiding officer shall nominate one member, who shall act as chairman of the committee; the member so nominated shall name a second, the second a third, and so on until the number of the committee is completed. The reports of all committees shall be made, in writing, and entered at length upon the minutes, unless when otherwise ordered by the society.

ARTICLE XVII.—*Awards of Merit.*

This society may award premiums to members, and others, for dissertations on subjects that may at any time be specified by the society at a stated meeting; also, for important improvements in mechanical dentistry and the manufacture of incorruptible teeth; and also for any suitable object. The question of preference, in all cases where more than one party is concerned, to be determined by the judgment of a special committee, appointed for that purpose. The unsuccessful article shall be at the disposal of the author or owner.

ARTICLE XVIII.

It shall be the duty of the society, at each stated meeting, to appoint at least two members to prepare dissertations upon some subject, (at the option of the members so appointed,) relative to the profession of dental surgery, to be read at the stated meeting; which when read, if upon a scientific or practical subject, shall be in order for discussion, under the usual parliamentary rules, which shall be determined by the President.

ARTICLE XIX.

Any member of this society who shall extol his own peculiar merits over those of a fellow practitioner, through the public prints, or make use of any secret nostrum or any patent instrument, or mode of practice whatever, which he is unwilling to communicate to the members of the profession, (recognized as such by this society,) shall be expelled.

ARTICLE XX.

No member of this society shall take a student for a less time than *two* years, unless he shall have studied dentistry with some other respectable practitioner, so as to make his time of pupilage equal to two years.

ARTICLE XXI.

A member violating any of the rules and regulations of the constitution and by-laws of this society shall be liable to impeachment.

Association adjourned till 4 o'clock, P. M.

*Four o'clock,* P. M.

Association called to order by the President.

Minutes of the morning session read and adopted.

Books required by the Association were ordered to be procured by the Recording Secretary.

On motion of Dr. J. D. White, the election for Examining Committee then took place, which resulted in the choice, for one year, of the following gentlemen:

DR. J. D. WHITE, *Chairman.*
J. M. HARRIS, DR. R. ARTHUR,
DR. ELI PARRY, STEPHEN T. BEALE.

A committee, consisting of Dr. Eli Parry, Dr. G. A. Planton, and W. J. Mullen, was then appointed, for the purpose of procuring a seal and certificate plate, and was required to report such devices for the seal as might seem to them most appropriate, at the next stated meeting of the Association.

The proceedings of the convention and Association were then ordered to be printed, in one daily and one weekly paper of the city of Philadelphia. Dr. R. Arthur, Dr. J. D. White, and C. C. Williams were appointed a publishing committee.

On motion of Dr. Eli Parry, seconded by Dr. J. D. White, Professor Chapin A. Harris, of Baltimore, and Dr. Daniel Harrington, of Philadelphia, were unanimously elected honorary members of the Association.

On motion of Dr. Parry, it was then

***Resolved,*** That Dr. Arthur be requested to deliver an opening address before the Association, at such time as may suit his convenience, and that, at such time, the President call a special meeting for the purpose, to attend which, the public shall be invited.

The Association then adjourned.

*Jan'y 20th,* 1846.—*Lecture Room of the Philada. Museum.*

The Association met specially, in accordance with a call from the President, by whom it was called to order.

The President then rose, and stated that Dr. Arthur having called on him, and informed him that he was prepared to deliver, before the Association, the opening address, and that the special meeting was called for the purpose.

Whereupon Dr. Arthur rose and addressed the meeting.

After the conclusion of the address, it was *Resolved*, on motion of J. M. Harris, that the address be recorded upon the minutes of the Association.

Then, on motion of J. D. White, a committee was appointed to wait on Dr. Arthur, for the purpose of requesting him to furnish a copy of his address for publication; the committee consisted of Dr. J. D. White, J. M. Harris, and F. Reinstein.

The Association then adjourned.

